# *Campylobacter* Colonization and Diversity in Young Turkeys in the Context of Gastrointestinal Distress and Antimicrobial Treatment

**DOI:** 10.3390/microorganisms11020252

**Published:** 2023-01-19

**Authors:** Margaret Kirchner, William G. Miller, Jason A. Osborne, Brian Badgley, Jeffrey Neidermeyer, Sophia Kathariou

**Affiliations:** 1 Department of Food, Bioprocessing, and Nutrition Sciences, North Carolina State University, Raleigh, NC 27695, USA; 2 Produce Safety and Microbiology Research Unit, Agricultural Research Service, US Department of Agriculture, Albany, CA 94710, USA; 3 Department of Statistics, North Carolina State University, Raleigh, NC 27695, USA; 4 School of Plant and Environmental Sciences, Virginia Tech, Blacksburg, VA 24061, USA

**Keywords:** turkeys, *Campylobacter*, antimicrobial resistance, multidrug resistance, irritable and crabby syndrome

## Abstract

Young turkeys are vulnerable to undifferentiated gastrointestinal distress, including “irritable and crabby syndrome” (ICS), which compromises flock performance and is typically treated with a combination of penicillin and gentamicin (P/G). However, the effects of ICS and P/G treatment on *Campylobacter* remain poorly understood. We investigated the impact of ICS and P/G treatment on *Campylobacter* levels and diversity in four flocks from three turkey farms. Cecum and jejunum samples were analyzed weekly from day of hatch to week 4–5. All four flocks became colonized with multidrug resistant (MDR) *Campylobacter jejuni* and *C. coli* by week 2–3, and two developed ICS. ICS and P/G treatment did not significantly impact total *Campylobacter* levels or strain genotypes but impacted species and antimicrobial resistance (AMR) profiles. One flock was raised under antibiotic-free (ABF) conditions while another flock at the same farm was raised conventionally. The ABF flock did not develop ICS while its counterpart did. However, *Campylobacter* strains, AMR profiles and sequence types were generally shared between these two flocks. Our findings suggest that ICS and P/G treatment impacted *Campylobacter* population dynamics in commercial young turkey flocks, and that ABF flocks may become readily colonized by MDR strains from non-ABF flocks at the same farm.

## 1. Introduction

*Campylobacter* is a leading cause of human bacterial gastroenteritis worldwide [1] and causes approximately 800,000 cases of disease (campylobacteriosis) annually in the United States alone [2]. Campylobacteriosis is also the most prevalent bacterial antecedent to Guillain-Barré Syndrome which can have long-term debilitating effects and contributes to reactive arthritis and other sequelae [1,3]. The majority of campylobacteriosis cases are caused by *C. jejuni* (90%) followed by *C. coli* [2]. The consumption and/or handling of raw or under-cooked poultry is a major risk factor for developing campylobacteriosis [4,5,6]. FoodNet, a foodborne pathogen monitoring system, found that *Campylobacter* is a leading cause of foodborne infections, with higher incidence in 2019 compared to 2016–2018 [7]. *Campylobacter* colonization has been extensively investigated in broilers but has not been as well characterized in turkeys [1,8,9].

*C. coli* and *C. jejuni* isolated from poultry often exhibit acquired a host of antimicrobial resistance (AMR) traits, notably resistance against fluoroquinolones and macrolides [10,11,12]. Such antimicrobial-resistant *Campylobacter* spp. have also been reported from young turkeys on brooder farms [10,13,14,15]. 

Young turkeys (brooders), from day of hatch to week 5, can be vulnerable to infection and gastrointestinal distress syndromes which are often attributed to an abnormal microbiota or “dysbiosis” in the birds [16,17,18]. The consequences of such gastrointestinal distress syndromes can be disastrous for bird health, final bird weight, and profitability of the farm operation [19,20]. One such syndrome frequently experienced in young turkey flocks in eastern North Carolina causes birds to appear restless and distressed, go off of feed and fail to gain weight and is herein referred to as “irritable crabby syndrome” (ICS). In the poultry industry these gastrointestinal syndromes are often treated with antimicrobials [21]. However, with heightened concerns about AMR among pathogenic microorganisms and antimicrobial use in animal agriculture [22,23,24], there is increasing interest in antibiotic-free (ABF) methods of rearing poultry [22,23,25,26]. 

To develop potential strategies for ICS mitigation, it would be critical to elucidate the potential microbiological underpinnings and implications of ICS and the accompanying treatment on the bacteria in the turkey gastrointestinal tract, including those that are of food safety concern, such as *Campylobacter*. This prompted the development of a partnership between our laboratory and veterinarians in the turkey industry which aimed to elucidate *Campylobacter* levels as well as gastrointestinal community composition and possible microbial community shifts associated with ICS and the corresponding treatment of young turkey flocks. The microbiome investigations will be described in a separate presentation. The objective of the present study was to characterize the prevalence and diversity of *Campylobacter* in brooder turkeys with or without ICS and administration of the corresponding antimicrobial treatment. To address these objectives, we investigated *Campylobacter* populations, AMR profiles, and genotypes at weekly intervals in four commercial turkey brooder flocks, including one produced under ABF standards. 

## 2. Materials and Methods

### 2.1. Turkey Flocks 

Due to the severe disease burden imposed by ICS in young turkeys discussed above, the partnering turkey company veterinarians upon consultation with the corresponding growers identified four commercial flocks (Table 1) for participation in the study. Three of these flocks were grown under conventional (CONV) industry standards (flocks 1, 2 and 3), while one of the flocks (flock 4) was grown under antibiotic-free (ABF) conditions (Table 1). All four flocks were obtained from the same breeder and placed at the farms as day-old birds. The design of the farms, size of turkey houses, general management and biosecurity practices were the same for all four flocks and followed industry standards. Flocks 1 and 2 were raised on separate farms (farms A and B, respectively), while flocks 3 and 4 were raised in different houses of the same farm (farm C). Farms A, B and C were operated by different growers under control of the same vertical integrator and were located in the same region (eastern North Carolina, USA). All flocks were fed a high-protein and high-starch starter feed composed of corn, wheat, and soymeal from day of hatch to 3.5 weeks old, at which time they received a lower-protein, lower-starch, and increased-fat diet for the remainder of the brooder period. The feed of CONV flocks (flocks 1, 2 and 3) included animal byproducts, ionophores and the coccidiostats monensin or lasalocid. Feed for flock 4 (ABF) contained the coccidiostat diclazuril and lacked animal byproducts, which were replaced with vegetable oil. Bird density was similar for the three CONV flocks (16,000–17,200/turkey house) while the ABF flock had lower bird density, with 9000 birds in the turkey house (Table 1). Flocks 2 and 3 developed ICS between weeks 3 and 4 and were treated with antibiotics (combination of penicillin and gentamicin, P/G) administered at therapeutic levels upon detection of ICS (Table 1). Flock 1 remained free of ICS but was treated with copper sulfate between weeks 4 and 5, possibly reflecting routine practices at farm A. 

### 2.2. Sample Collection, Campylobacter Isolation and Enumeration

As indicated above, the four commercial flocks (Table 1) were included in the study by turkey company veterinarians upon consultation with the growers for farms A, B and C. At placement (day-old birds) and once each week, 10 birds from each flock were randomly chosen by the turkey company veterinarians and euthanized at the company’s facility following the company’s animal welfare guidelines, as described before [14]. A segment (approx. 3–4 cm) of the jejunum and one cecum from each bird were placed in separate bags and labeled to indicate bird, date, flock number and flock age. Samples were shipped by the company veterinarians to our laboratory at North Carolina State University overnight on ice and kept at 4 °C until processing, typically within 4 h. The cecum and jejunum samples of each bird were processed individually from weeks 2–5. The day of hatch and week 1 samples were pooled for processing because it was unlikely that they would yield *Campylobacter* [10,15,27,28]. For early time points (day of hatch to week 3), samples were also enriched for *Campylobacter* as previously described [29]. To detect and enumerate *Campylobacter*, 0.1 g of sample was suspended in 1.0 mL of Mueller-Hinton broth (MHB) (Becton Dickinson, Sparks, MD, USA), diluted serially in MHB, and 10 μL of the serial dilutions were spotted onto blood-free modified charcoal cefoperazone desoxycholate agar (mCCDA; Oxoid, Hampshire, UK). Selected dilutions were also plated (100 μL) onto mCCDA. Plates were incubated under microaerobic conditions generated by a GasPak EZ Campy sachet (Becton, Dickinson and Co., Sparks, MD, USA) for 48 h at 42 °C. A minimum of one isolate from each positive bird was purified from mCCDA at each time point by streaking onto MHA (Mueller-Hinton broth with 1.2% agar). All isolates were stored at −80 °C as previously described [15,30]. 

### 2.3. Determination of Campylobacter Species, AMR profiles and Genotypes 

The *Campylobacter* species of each isolate was determined by multiplex PCR using *hip* (5′-ATG ATG GCT TCT TCG GAT AG-3′ and 5′-GCT CCT ATG CTT ACA ACT GC-3′) and *ceu* (5′-GAT TTT ATT ATT TGT AGC AGC G-3′ and 5′-TCC ATG CCC TAA GAC TTA ACG-3′) primers to identify *C. jejuni* and *C. coli*, respectively, as previously described [15,30]. Briefly, reactions were performed using X-Taq DNA polymerase (Fisher, Fair Lawn, NJ, USA) in 25 mL with 0.5 μL of genomic DNA as a template. The reaction conditions included an initial denaturation at 95 °C for 5 min, followed by 30 cycles of 95 °C for 1 min, 50 °C for 1 min, and 72 °C for 2 min, with a final extension at 72 °C for 5 min. AMR profiles were determined as previously described on antibiotic-amended MHA [30]. Briefly, isolates were spotted (3.5 μL) in duplicate on MHA amended with tetracycline (16 μg/mL), streptomycin (64 μg/mL), erythromycin (8 μg/mL), kanamycin (64 μg/mL), nalidixic acid (32 μg/mL), ciprofloxacin (4 μg/mL), or gentamicin (50 μg/mL). MHA without added antibiotics and the pan-sensitive *C. jejuni* ATCC 33560 were used each time for quality assurance. Plates were incubated for 48 h at 42 °C under microaerobic conditions, and resistance was determined based on the visual assessment for confluent growth on both spots. For genotyping, multilocus sequence typing (MLST) was done using the primers *tktFN/tktRN* [31] together with primers *aspAF1/aspAR1, atpAF/atpAR, glnAF/glnAR, gltAF/gltAR, glyAF/glyAR* and *pgmF1/pgmR1* [32]. Each amplification used 50 ng genomic DNA and 50 pmol each primer under the following conditions: 30 s at 94 °C, 30 s at 53 °C and 2 min at 72 °C (30 cycles) [32]. Sequencing reactions were performed using the same amplification primers, as described [32]. Alleles and sequence types were assigned using MLSTparser [32] and a database of *C. jejuni* and *C. coli* MLST alleles and STs. Novel allelic profiles were submitted to PubMLST (https://pubmlst.org/organisms/campylobacter-jejunicoli; accessed on 5 December 2022) for ST assignment. Minimum spanning trees (MSTs) were prepared using BioNumerics, as previously described [31]. 

### 2.4. Statistical Analysis

A t-test was used to compare species/AMR combinations before and after antibiotics within each flock and to compare the prevalence of species in the cecum and jejunum. A two-way ANOVA was used to investigate the prevalence and enumeration of *Campylobacter* within and between flocks. 

## 3. Results and Discussion

### 3.1. Campylobacter Colonization Varies among Individuals and Intestinal Site

**Figure 1:**
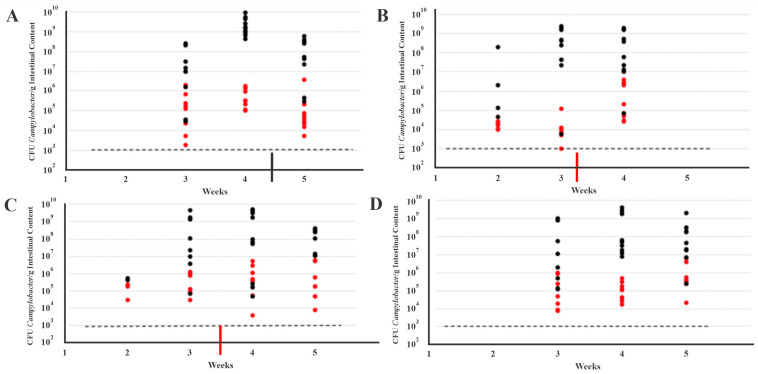
*Campylobacter* load within the cecum and jejunum from the four turkey flocks. The average *Campylobacter* load of the ten sampled birds from (**A**) flock 1, (**B**) 2, (**C**) 3, and (**D**) 4 are shown for each time point starting with week 1. Red and black dots represent CFU/g counts from the jejunum and cecum, respectively. A thick black line and a thick red line along the x axis indicate the time of administration of copper sulfate and penicillin/gentamicin, respectively. The limit of detection (horizontal black dotted lines) was 1.00 × 10^3^ CFU/g in the cecum and 1.00 × 10^1^ CFU/g in the jejunum.

All four turkey brooder flocks became positive for *Campylobacter* by week 2 (flocks 2 and 3) or 3 (flocks 1 and 4) (Table 1). Previous samples were negative by direct plating as well as by enrichment. Interestingly, only the flocks that developed ICS had detectable *Campylobacter* in week 2 (Table 1). This opens up the possibility that the flocks colonized earlier were pre-disposed to ICS or vulnerable due to stress, or that *Campylobacter* could be a contributing factor for development of ICS. No noticeable changes in the levels of *Campylobacter* in the cecum or the jejunum were noted in flocks 2 and 3 after ICS and P/G treatment, while in flock 1 some decreases were noted after copper sulfate treatment, especially in the cecum (Figure 1 and Supplementary Table S1).

For flocks 2 and 3 only some of the birds were positive in the first week of *Campylobacter* detection (week 2) and the *Campylobacter* levels in the few positive birds were generally below 10^6^ CFU/g in the cecum (Supplementary Table S1). In contrast, for flocks 1 and 4 that did not develop ICS the birds were all positive in the very week that *Campylobacter* was detected, i.e., week 3, and the *Campylobacter* CFU/g cecum exceeded 10^6^ for most birds (Supplementary Table S1). These findings indicate a surprisingly sudden onset of high-level *Campylobacter* colonization in ICS-free flocks, while in those that developed ICS the onset was more gradual, similarly to what was described previously for turkey colonization with *Campylobacter* [28]. It is possible that the ICS-free flocks were also colonized in week 2 but the numbers were too low for the detection methods that we employed, even with the inclusion of enrichments. In the weeks that followed, the birds from all four flocks were all positive with the *Campylobacter* levels in the cecum ranging between 1 × 10^7^ and 1 × 10^9^ CFU/g (with only few occasional exceptions (Supplementary Table S1), as reported previously [28].

Even though most birds tested positive for *Campylobacter* in the cecum and jejunum after the initial detection, noticeable bird-to-bird variation in CFU *Campylobacter*/g cecal or jejunal content was observed (Figure 1 and Supplementary Table S1). A low level of *Campylobacter* in the cecum was not always accompanied with a low level in the jejunum (Supplementary Table S1). *Campylobacter* CFU/g values in the cecum (maximum 10^9^ CFU/g) were at least 3-log higher than in the jejunum (maximum 10^6^ CFU/g) (Figure 1). This is in agreement with a previous study that documented lower *Campylobacter* colonization in the jejunum versus the cecum [28]. The difference in colonization between intestinal sites in this study ranged between 2–3 logs (Supplementary Table S1). 

As indicated above all birds in the ABF flock were positive starting with week 3, and the average CFU *Campylobacter*/g of cecal content was similar to the levels noted with the other three flocks (Figure 1). Thus, the level of colonization was overall similar and comparable across all flocks regardless of management (ABF vs. conventional) or treatment. It appears that the ABF production practices did not affect *Campylobacter* levels in the cecum and jejunum, as previously demonstrated in European organic flocks [33,34]. However, it is worthy of note that flock 4 (ABF flock) yielded *Campylobacter*-positive samples one week later than flock 3, which was housed at the same farm and grown under conventional industry practices (Table 1). Later onset of *Campylobacter* colonization in this flock could be associated in part with the slower weight gain generally observed with ABF turkeys, similarly to previous findings in birds grown without antibiotics [19,21,35]. 

*Campylobacter* isolates (*n* = 372) included 90, 90, 96 and 96 isolates from flocks 1, 2, 3 and 4, respectively (Table 2). All isolates were identified as either *C. coli* or *C. jejuni* and *C. coli* was most frequently isolated overall (57.8%), except for flock 3 where *C. coli* and *C. jejuni* were present at about equal proportions (Table 2). Interestingly, *C. coli* was overall significantly more common (*p* < 0.05) in the cecum (86.3%) than in the jejunum (13.2%) while *C. jejuni* was significantly more common (*p* < 0.05) in the jejunum (74.4%) than in the cecum (25.6%) (Table 2). Flock 2 was the only flock that had more *C. coli* (62.5%) than *C. jejuni* (37.5%) in the jejunum (Table 2). The mechanisms underlying the apparent predilection of *C. jejuni* for the jejunum, and *C. coli* for the cecum, remain to be elucidated. Strong preferential association of *C. coli* and *C. jejuni* with the cecum and the jejunum, respectively, has been repeatedly noted before in our laboratory in assessments of gastrointestinal samples from turkeys and other birds (chickens, guineafowl) grown commercially (R. M. Siletzky and S. Kathariou, unpublished findings).

### 3.2. Diversity of Campylobacter AMR Profiles and MLST Sequence Types Vary by Flock 

By combining both species and AMR profile, 12 (six each in *C. coli* and *C. jejuni*) total distinct species/AMR combinations were identified, including eight in flock 1 and six each in flocks 2–4 (Table 2). MLST was employed to genotype isolates from week 2 (*n* = 3), 3 (*n* = 15), 4 (*n* = 22), and 5 (*n* = 13) from all four flocks. One isolate for each individual species/AMR combination within each flock was chosen for MLST, typically from the cecum. When specific species/AMR combinations were detected in multiple weeks, isolates from multiple time points were included (Supplementary Table S2). 

Multidrug resistance (MDR), i.e., resistance to three or more antimicrobial classes, was seen in 90% of the isolates, and all isolates were resistant to tetracycline. Erythromycin resistance was only seen among *C. coli* isolates, which is consistent with previous studies of *Campylobacter* from turkeys from this region [14,30,36]. AMR profile designations were created for each isolate by using the first letter of each antibiotic the isolate was resistant to, except for the (fluoro)quinolones nalidixic acid and ciprofloxacin, which were represented by “Q”. The two most predominant species/AMR profiles were *C. coli* resistant to all tested antimicrobials i.e., tetracycline (T), streptomycin (S), erythromycin (E), kanamycin (K), gentamicin (G), nalidixic acid and ciprofloxacin (Q), i.e., ccTSEKQG, and *C. jejuni* resistant to all tested antimicrobials except for erythromycin, i.e., cjTSKQG, accounting for 37.6% and 34.4%, respectively of the 372 isolates (Table 2). The AMR profiles TSKQG, TSKQ, TK, and TKQ were only found in *C. jejuni* and none of the *C. jejuni* isolates exhibited resistance to erythromycin while erythromycin resistance was commonly encountered in *C. coli*, similarly to previous studies of *Campylobacter* from turkeys in eastern North Carolina [14,15,30,36]. 

**Figure 2:**
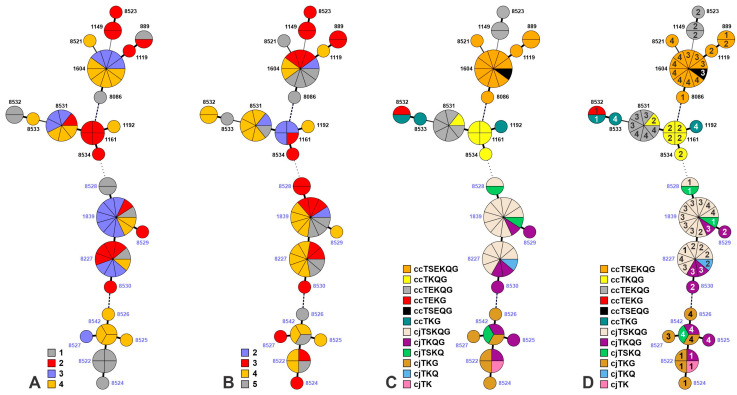
Minimum spanning tree of the *Campylobacter* MLST sequence types. Minimum spanning tree showing the clustering of the MLST-based STs of 69 *C. coli* and *C. jejuni* isolates. Each tree is colored based on (**A**) flock, (**B**) week, (**C**) AMR profile, or (**D**) AMR profile by color and flock by number. The tree was created in BioNumerics (v. 7.6). Each ST is represented by a circle with each segment in the circle corresponding to an individual isolate. Sequence types in blue font are *C. jejuni* while those in black font are *C. coli*. Thick short lines connecting STs indicate single-locus differences; thin lines indicate two-locus differences; a longer, thinner line indicates a three-locus difference; and black dotted lines represent > four-allele differences. The gray dotted line between STs 8528 and 8534 separates *C. jejuni* STs in the bottom (blue font STs) from *C. coli* STs in the top (black font STs).

MLST analysis indicated that overall *C. coli* and *C. jejuni* exhibited similar diversity and 24 STs were totally identified, 11 and 13 in *C. jejuni* and *C. coli*, respectively (Table 3 and Figure 2A). Several of these STs have been repeatedly encountered in other studies of turkeys from eastern North Carolina [14,30,31,36]. However, 15 of the 24 STs were novel, including six in *C. coli* and nine in *C. jejuni* (Table 3). Of the 15 novel STs, eight were closely related (maximum of 2 allelic differences) to known STs identified in the four flocks and six *C. jejuni* STs (8522, 8524, 8525, 8526, 8527 and 8542) were closely related to each other but not to a known ST within the flocks (Figure 2A). The two most common STs in the *C. coli* isolates were STs 1604 (*n* = 10) and 8531 (*n* = 7), a novel ST closely related to ST-1161, 1192, 8534, and 8533, and the two most common *C. jejuni* STs were 8227 (*n* = 9) and 1839 (*n* = 11). These dominant *C. coli* and *C. jejuni* STs were shared among different flocks and encountered in birds of diverse ages (Figure 2A,B). Novel STs were found in all flocks with the highest number found in flock 4 (*n* = 6) and the lowest in flock 3 (*n* = 2) (Table 3).

Farms A (flock 1), B (flock 2) and C (flocks 3 and 4) were within 37 miles of each other and managed by the same company. This overlap and relative proximity may account for the similarity of STs across the different flocks and suggests that certain *C. jejuni* and *C. coli* STs may persist in commercial turkey production in eastern North Carolina (Figure 2A, Table 3).

The apparent similar levels of diversity within *C. jejuni* and *C. coli* is in contrast with findings with isolates from three turkey farms in Ohio, where higher diversity was noted in *C. coli* [10]. The gastrointestinal site (e.g., cecum vs. jejunum) from which the isolates originated was not reported in this previous study [10]. The fact that isolates both from jejunum and cecum were included in the current study, and *C. jejuni* was found to be noticeably more common in the jejunum, may have enhanced the opportunity to survey diverse strains of both species colonizing the birds. It is also noteworthy that none of the 11 *C. jejuni* STs in our study, and only two of the 13 *C. coli* STs (STs 889 and 1119, encountered in just three of our *C. coli* strains) overlapped with those from the turkeys from the farms in Ohio [10], suggesting noticeable regional diversity in the genotypes of *C. jejuni* and *C. coli* colonizing commercial turkeys. The underlying reasons remain to be elucidated but may be related to management practices. 

MLST identified two main clusters in *C. coli* as well as two main clusters in *C. jejuni* (Figure 2A). These clusters in *C. jejuni* tended to form around AMR profiles, with cjTSKQG and cjTKG belonging to the same or closely related STs (Figure 2C). Most species/AMR combinations were of the same or closely related STs (Figure 2C). Two notable exceptions were the *C. jejuni* profiles cjTKQG and cjTSKQ, which were identified in two clearly-distinct clusters, each consisting of highly related STs (Figure 2C). 

### 3.3. Penicillin and Gentamicin Treatment Are Associated with a Shift in Certain Campylobacter AMR Profiles

**Figure 3:**
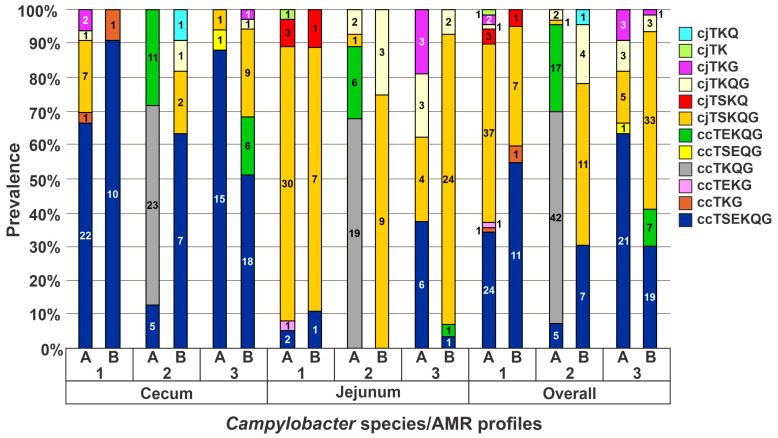
*Campylobacter* species and AMR profile pre- and post-treatment in isolates from the cecum and the jejunum of the three turkey flocks. Distribution of *Campylobacter* species/AMR profiles among isolates from the cecum and jejunum before (**A**) and after (**B**) treatment of the flocks which underwent treatment with copper sulfate (flock 1) or penicillin/gentamicin (flocks 2 and 3).

As indicated earlier, flocks 2 and 3 developed ICS between weeks 3 and 4 and were P/G-treated (Table 1), allowing the opportunity to assess potential impacts of ICS and P/G treatment on *Campylobacter* species and strain distributions (Figure 3). In flock 2, ccTKQG was a species/AMR profile unique to this flock. This profile was dominant in the cecum and jejunum of flock 2 in week 2 and also encountered in week 3 (Table 2). Another species/AMR profile, ccTEKQG, first appeared in flock 2 in week 3, composing a majority of the isolates in the cecum (11/20) and jejunum (6/8). Interestingly, however, neither ccTKQG nor ccTEKQG were detected in flock 2 after P/G treatment (Table 2, Figure 3). Instead, after P/G treatment the dominant species/AMR profiles in flock 2 shifted completely to ccTESKQG and cjTSKQG in the cecum and jejunum, respectively (Table 2, Figure 3). Treatment with P/G was followed by a significant shift in species/AMR profiles in flock 2 with a decrease in ccTKQG and ccTEKQG (*p* < 0.0001) and a concomitant increase in cjTSKQG (*p* < 0.0001).

In flock 3, ccTEKQG was detected in flock 3 only after P/G treatment (Table 2, Figure 3). The ccTEKQG isolates identified in this flock as well as those in the ABF flock 4 housed at the same farm were ST-8531, while ccTEKQG from flock 2 had the markedly distinct STs 8523 and 1149. which actually belonged to a different *C. coli* cluster (Figure 2D). These findings suggest that ccTEKQG was independently introduced into the farm that housed flock 2 and the one that housed flocks 3 and 4, and might not be affected by the antibiotics administered to flock 3. Similarly to flock 2, a significant overall increase in cjTSKQG (*p* = 0.0005) after antimicrobial treatment was seen in flock 3. The jejunum of flock 3 birds at week 3 also appeared to have greater strain diversity than in weeks 4 or 5, after P/G had been administered. Even though cjTSKQG and two other profiles (cjTKQG, cjTKG) were detected in similar proportions in week 3 prior to treatment, all but one post-treatment isolates from week 4, and all those from week 5, were cjTSKQG (Table 2, Figure 3). The post-treatment increases in cjTSKQG in both flocks 2 and 3 raise the possibility that frequent P/G treatment of young turkeys for ICS and similar gastrointestinal disturbances may contribute to the apparent dissemination of multidrug resistant cjTSKQG strains in eastern North Carolina observed here and in other studies [14,36]. 

### 3.4. Impacts of Copper Sulfate Treatment on Campylobacter Diversity 

Flock 1 was reported to be healthy throughout the study period but was treated with copper sulfate, an antimicrobial, between weeks 4 and 5. The most common species/AMR combinations in this flock were ccTSEKQG and cjTSKQG (Table 2). ST-8086, only found in flock 1, and ST-889 comprised the ccTSEKQG isolates from flock 1 while STs 8227 and 8528 comprised the cjTSKQG isolates (Figure 2D). Four other *C. jejuni* and *C. coli* STs were only seen in flock 1 and were closely related to at least one other ST (Figure 2A). Other species/AMR combinations were transient (i.e., detected only during one week), including ccTEKG and cjTK, which were only detected in flock 1 (Table 2). Both of these unique species/AMR combinations had novel STs. 

Before copper treatment of flock 1, ccTSEKQG was dominant in the cecum and cjTSKQG was dominant in the jejunum with four other species/AMR combinations also encountered both in the cecum and the jejunum (Table 2, Figure 3). After copper treatment, ccTSEKQG and cjTSKQG remained dominant in the cecum and jejunum but the number of additional species/AMR combinations decreased from five to two in the cecum and from five to three in the jejunum (Table 2, Figure 3). Even though the small numbers of STs did not allow statistical assessments of significance, the findings suggest the possibility that copper sulfate treatment may be accompanied with decreased diversity in species/AMR profile combinations. 

### 3.5. Campylobacter Strains Are Largely Similar between ABF and Non-ABF Flocks on the Same Farm 

Flocks 3 (ABF) and 4 (non-ABF) were raised on the same farm (Table 1). This allowed us to compare *Campylobacter* diversity, colonization, and genotypes between a non-ABF flock that contracted ICS and was treated and a flock that was raised under ABF conditions and did not contract ICS. The flocks were highly comparable in respect to *Campylobacter* diversity, sharing the same dominant species/AMR combinations, ccTSEKQG and cjTSKQG, throughout the study and many of the same transient species/AMR combinations (Table 2). The exceptions were ccTSEQG and ccTKG, detected only in flock 3 and 4, respectively. The presence of MDR observed here was markedly higher than observed in previous studies of poultry flocks raised without antibiotics [25,37,38]. This could be attributed to the ABF flock being raised on the same farm as flocks that were colonized with MDR *Campylobacter* strains. Flocks 3 and 4 shared two *C. coli* STs, 1604 and 8531, and two *C. jejuni* STs, 8227 and 1839 (Figure 2D). There were several STs only found in flock 4, including STs 8542, 8525 and 8526 in *C. jejuni* and STs 8521, 8533 and 1192 in *C. coli* (Table 3). These *C. jejuni* STs were all closely related to each other and encompassed cjTKQG, cjTSKQ, and cjTKG (Figure 2D); however, among *C. coli,* ccTSEKQG with ST-8521 was only found in flock 4, and the two ccTKG isolates in flock 4 had distinct STs (1192 and 8533) not found in flock 3 (Figure 2D).

Three of the transient species/AMR combinations, cjTKQG, cjTKG, and ccTEKQG, which made up 30.8%, 19.2% and 38.5%, respectively, of all non-major species/AMR combinations, appeared in flock 3 either before or concurrently with flock 4 (Table 2). This suggests that they were introduced to farm C where flocks 3 and 4 were housed and were not consistently present in young turkeys at other farms. Since non-ABF flocks were concurrently raised in the farm (farm C) that housed flock 4, the environment would be expected to be similar to commercial farms operating under standard industry practices. Commercial turkey farms in this region were previously found to be colonized with MDR *C. jejuni* and *C. coli*. [13,14,15,36]. This established presence of MDR strains in the region could also explain why the species/AMR combinations found in flocks 3 and 4 were so comparable (Table 2). 

## 4. Conclusions

Our findings were based on the analysis of four brooder turkey flocks in eastern North Carolina, a major turkey-producing region in the United States. Clearly, additional studies are needed with larger numbers of turkey flocks, and in diverse regions. It would be also desirable to monitor the birds subsequent to the brooder period, which was not feasible in our study due to the logistics of turkey production where the brooders would be transported to different and often distantly located grow-out farms. Nonetheless, our findings indicate that ICS and P/G treatment noticeably impacted *Campylobacter* population dynamics and diversity in commercial young turkey flocks. The prevalence of certain *C. jejuni* and *C. coli* strains with multidrug resistance profiles increased significantly the week after penicillin/gentamicin treatment in both ICS-afflicted flocks, raising the possibility that gastrointestinal distress episodes in young turkeys and the accompanying antimicrobial treatment may contribute the high prevalence of such multidrug-resistant strains in commercial turkey production. Thus, flock management strategies to mitigate gastrointestinal distress in young turkeys may also contribute to reductions in the prevalence of multidrug-resistant *Campylobacter* in the flocks. Another novel finding was the significant association of *C. coli* and *C. jejuni* with the turkey cecum and jejunum, respectively. Thus, accurate surveillance of *Campylobacter* in turkey flocks may benefit from inclusion of both of these gastrointestinal tract sites. Lastly, the similarity of species/AMR profiles across flocks, especially flocks 3 (conventional) and 4 (ABF) which were housed on the same farm, serves to underscore that in-flock management practices are not always sufficient to mitigate the high prevalence of antimicrobial resistance in *Campylobacter* from young turkeys. The environment in which the young turkeys are raised, including proximity to other flocks, along with management practices and the persistence of AMR genes, can all contribute to the AMR profiles of *C. jejuni and C. coli* that colonize the flocks. 

## Figures and Tables

**Table 1:** Turkey flocks investigated in this study.

Dates ^1^	Flock	Farm	Rearing Method ^2^	Birds per House	Duration of Life (Weeks)	Initial *Campylobacter* Colonization	ICS	Treatment ^3^	Time of Treatment
19 April 2016–25 May 2016	1	A	CONV	16,000	5	Week 3	No	Cu	Weeks 4–5
19 April 2016–18 May 2016	2	B	CONV	17,200	4	Week 2	Yes	P/G	Weeks 3–4
26 April 2016–1 June 2016	3	C	CONV	17,000	5	Week 2	Yes	P/G	Weeks 3–4
26 April 2016–1 June 2016	4	C	ABF	9000	5	Week 3	No	N/A	N/A

^1^ Date format is Month / Day/ Year. ^2^ “CONV” designates conventional flock rearing practices and “ABF” designates antibiotic-free rearing practices, as detailed in the Materials and Methods. ^3^ “Cu”, flock treated with copper sulfate, “P/G”, flock treated with penicillin and gentamicin. “N/A” (non-applicable) indicates a flock for which no known antimicrobial treatment was administered.

**Table 2:** Distribution of *Campylobacter* species and AMR profiles in the cecum and jejunum from the four turkey flocks. *Campylobacter* Species/AMR profiles ^1^.

Flock	Week ^2^	Intestinal Site	ccTSEKQG	ccTKG	ccTEKG	ccTKQG	ccTSEQG	ccTEKQG	Total Cc	cjTSKQG	cjTSKQ	cjTKQG	cjTKG	cjTK	cjTKQ	Total Cj
1	1	Cecum	0	0	0	0	0	0	0	0	0	0	0	0	0	0
		Jejunum	0	0	0	0	0	0	0	0	0	0	0	0	0	0
	2	Cecum	0	0	0	0	0	0	0	0	0	0	0	0	0	0
		Jejunum	0	0	0	0	0	0	0	0	0	0	0	0	0	0
	3	Cecum	13	0	0	0	0	0	13	7	0	0	1	0	0	8
		Jejunum	1	0	0	0	0	0	1	22	3	0	0	1	0	26
	4	Cecum	9	1	0	0	0	0	10	0	0	1	1	0	0	2
		Jejunum	1	0	1	0	0	0	2	8	0	0	0	0	0	8
	5 *	Cecum	10	1	0	0	0	0	11	0	0	0	0	0	0	0
		Jejunum	1	0	0	0	0	0	1	7	1	0	0	0	0	8
2	1	Cecum	0	0	0	0	0	0	0	0	0	0	0	0	0	0
		Jejunum	0	0	0	0	0	0	0	0	0	0	0	0	0	0
	2	Cecum	0	0	0	19	0	0	19	0	0	0	0	0	0	0
		Jejunum	0	0	0	17	0	0	17	0	0	0	0	0	0	0
	3	Cecum	5	0	0	4	0	11	20	0	0	0	0	0	0	0
		Jejunum	0	0	0	2	0	6	8	1	0	2	0	0	0	3
	4 *	Cecum	7	0	0	0	0	0	7	2	0	1	0	0	1	4
		Jejunum	0	0	0	0	0	0	0	9	0	3	0	0	0	12
3	1	Cecum	0	0	0	0	0	0	0	0	0	0	0	0	0	0
		Jejunum	0	0	0	0	0	0	0	0	0	0	0	0	0	0
	2	Cecum	5	0	0	0	0	0	5	1	0	0	0	0	0	1
		Jejunum	3	0	0	0	0	0	3	2	0	0	0	0	0	2
	3	Cecum	10	0	0	0	1	0	11	0	0	0	0	0	0	0
		Jejunum	3	0	0	0	0	0	3	2	0	3	3	0	0	8
	4 *	Cecum	8	0	0	0	0	6	14	0	0	0	1	0	0	1
		Jejunum	0	0	0	0	0	1	1	7	0	2	0	0	0	9
	5 *	Cecum	10	0	0	0	0	0	10	9	0	1	0	0	0	10
		Jejunum	1	0	0	0	0	0	1	17	0	0	0	0	0	17
4	1	Cecum	0	0	0	0	0	0	0	0	0	0	0	0	0	0
		Jejunum	0	0	0	0	0	0	0	0	0	0	0	0	0	0
	2	Cecum	0	0	0	0	0	0	0	0	0	0	0	0	0	0
		Jejunum	0	0	0	0	0	0	0	0	0	0	0	0	0	0
	3	Cecum	25	0	0	0	0	0	25	0	0	0	0	0	0	0
		Jejunum	2	0	0	0	0	0	2	10	0	0	0	0	0	10
	4	Cecum	12	1	0	0	0	0	13	0	0	0	0	0	0	0
		Jejunum	1	0	0	0	0	2	3	9	0	2	0	0	0	11
	5	Cecum	11	1	0	0	0	1	13	0	1	0	0	0	0	1
		Jejunum	2	0	0	0	0	0	2	15	0	0	1	0	0	16
Total		Cecum	125	4	0	23	1	18	171	19	1	3	3	0	1	27
		Jejunum	15	0	1	19	0	9	44	109	4	12	4	1	0	130
		Overall	140	4	1	42	1	27	215	128	5	15	7	1	1	157

^1^ The isolate identifiers consist of the species designation (cc and cj indicating *C. coli* and *C. jejuni*, respectively) followed by the AMR profile determinized by assessing resistance to tetracycline (T), streptomycin (S), erythromycin (E), gentamicin (G), kanamycin (K), and the (fluoro)quinolones nalidixic acid and ciprofloxacin (Q), with Q indicating resistance to both nalidixic acid and ciprofloxacin. For instance, *C. coli* resistant to all tested antibiotics would be denoted as a ccTSEKQG, *C. jejuni* resistant to all tested antibiotics except erythromycin would be designated cjTSKQG, and *C. coli* resistant to tetracycline, kanamycin and gentamicin but none of the other tested antimicrobials would be designated ccTKG. Gray shadowing indicates detection of the indicated species/AMR profiles in the number of isolates shown. ^2^ * indicates post-treatment.

**Table 3:** *Campylobacter jejuni* and *C. coli* sequence types identified in the four turkey flocks.

ST ^1^	Clonal Complex ^1^	Species	AMR Profiles ^2^	Number of Isolates	Flock 1	Flock 2	Flock 3	Flock 4
889	828	*C. coli*	TSEKQG	2	1	1	0	0
1119	828	*C. coli*	TSEKQG	1	0	1	0	0
1149	828	*C. coli*	TEKQG	2	0	2	0	0
1161	1150	*C. coli*	TKQG	4	0	4	0	0
1192	1150	*C. coli*	TKG	1	0	0	0	1
1604	828	*C. coli*	TSEKQG (*n* = 9), TSEQG (*n* = 1)	10	0	0	4	6
8086	828	*C. coli*	TSEKQG	1	1	0	0	0
**8521**	828	*C. coli*	TSEKQG	1	0	0	0	1
**8523**	828	*C. coli*	TEKQG	1	0	1	0	0
**8531**	1150	*C. coli*	TEKQG (*n* = 6), TKQG (*n* = 1)	7	0	1	3	3
**8532**	1150	*C. coli*	TEKQ (*n* = 1), TKG (*n* = 1)	2	2	0	0	0
**8533**	1150	*C. coli*	TKG	1	0	0	0	1
**8534**	1150	*C. coli*	TKQG	1	0	1	0	0
1839	Unknown	*C. jejuni*	TSKQG (*n* = 9), TKQG (*n* = 1), TSKQ (*n* = 1)	11	1	1	7	2
8227	Unknown	*C. jejuni*	TSKQG (*n* = 6), TKQG (*n* = 2), TKQ (*n* = 1)	9	1	4	3	1
**8522**	353	*C. jejuni*	TKG (*n* = 2), TKQG (*n* = 1), TK (*n* = 1)	4	4	0	0	0
**8524**	Unknown	*C. jejuni*	TKG	1	1	0	0	0
**8525**	353	*C. jejuni*	TKQG	1	0	0	0	1
**8526**	Unknown	*C. jejuni*	TKG	1	0	0	0	1
**8527**	353	*C. jejuni*	TKG	1	0	0	1	0
**8528**	Unknown	*C. jejuni*	TSKQG (*n* = 1), TSKQ (*n* = 1),	2	2	0	0	0
**8529**	Unknown	*C. jejuni*	TKQG	1	0	1	0	0
**8530**	Unknown	*C. jejuni*	TKQG	1	0	1	0	0
**8542**	353	*C. jejuni*	TKQG (*n* = 1), TSKQ (*n* = 1), TKG (*n* = 1)	3	0	0	0	3

^1^ Sequence types (STs) were determined by multilocus sequence typing (MLST) as described in Materials and Methods. Designations in bold indicate novel STs. “Unknown” CC indicates singleton STs not currently known to belong to a known CC. ^2^ AMR profile identifiers are as described for Table 2. When multiple AMR profiles were encountered among isolates of the same ST, the numbers of isolates with each profile are in parentheses.

## Data Availability

Data for this manuscript can be found in the Supplementary Materials. Additional information regarding novel or existing MLST alleles and sequence types can be obtained from PubMLST (https://pubmlst.org/, accessed on 5 December 2022).

## Supplementary Materials

The following supporting information can be downloaded at: https://www.mdpi.com/article/10.3390/microorganisms11020252/s1, Supplementary Table S1: *Campylobacter* content in the cecum and jejunum of individual birds; Supplementary Table S2: *Campylobacter* isolates typed by MLST.

## References

[1] Kaakoush N.O., Castano-Rodriguez N., Mitchell H.M., Man S.M. (2015). Global epidemiology of *Campylobacter* infection. Clin. Microbiol. Rev..

[2] Scallan E., Hoekstra R.M., Angulo F.J., Tauxe R.V., Widdowson M.A., Roy S.L., Jones J.L., Griffin P.M. (2011). Foodborne illness acquired in the United States—Major pathogens. Emerg. Infect. Dis..

[3] Nyati K.K., Nyati R. (2013). Role of *Campylobacter jejuni* infection in the pathogenesis of Guillain-Barre syndrome: An update. Biomed. Res. Int..

[4] Buettner S., Wieland B., Staerk K.D., Regula G. (2010). Risk attribution of *Campylobacter* infection by age group using exposure modelling. Epidemiol. Infect..

[5] Kittl S., Korczak B.M., Niederer L., Baumgartner A., Buettner S., Overesch G., Kuhnert P. (2013). Comparison of genotypes and antibiotic resistances of *Campylobacter jejuni* and *Campylobacter coli* on chicken retail meat and at slaughter. Appl. Environ. Microbiol..

[6] Mughini Gras L., Smid J.H., Wagenaar J.A., de Boer A.G., Havelaar A.H., Friesema I.H., French N.P., Busani L., van Pelt W. (2012). Risk factors for campylobacteriosis of chicken, ruminant, and environmental origin: A combined case-control and source attribution analysis. PLoS ONE.

[7] Tack D.M., Ray L., Griffin P.M., Cieslak P.R., Dunn J., Rissman T., Jervis R., Lathrop S., Muse A., Duwell M. (2020). Preliminary incidence and trends of infections with pathogens transmitted commonly through food—Foodborne Diseases Active Surveillance Network, 10 U.S. Sites, 2016–2019. MMWR Morb. Mortal. Wkly. Rep..

[8] Kalupahana R.S., Kottawatta K.S., Kanankege K.S., van Bergen M.A., Abeynayake P., Wagenaar J.A. (2013). Colonization of *Campylobacter* spp. in broiler chickens and laying hens reared in tropical climates with low-biosecurity housing. Appl. Environ. Microbiol..

[9] Newell D.G., Fearnley C. (2003). Sources of *Campylobacter* colonization in broiler chickens. Appl. Environ. Microbiol..

[10] Kashoma I.P., Kumar A., Sanad Y.M., Gebreyes W., Kazwala R.R., Garabed R., Rajashekara G. (2014). Phenotypic and genotypic diversity of thermophilic *Campylobacter* spp. in commercial turkey flocks: A longitudinal study. Foodborne Pathog. Dis..

[11] Luangtongkum T., Jeon B., Han J., Plummer P., Logue C.M., Zhang Q. (2009). Antibiotic resistance in *Campylobacter*: Emergence, transmission and persistence. Future Microbiol..

[12] Lutgen E.M., McEvoy J.M., Sherwood J.S., Logue C.M. (2009). Antimicrobial resistance profiling and molecular subtyping of *Campylobacter* spp. from processed turkey. BMC Microbiol..

[13] Lee B.C., Reimers N., Barnes H.J., D’Lima C., Carver D., Kathariou S. (2005). Strain persistence and fluctuation of multiple-antibiotic resistant *Campylobacter coli* colonizing turkeys over successive production cycles. Foodborne Pathog. Dis..

[14] Niedermeyer J.A., Ring L., Miller W.G., Genger S., Lindsey C.P., Osborne J., Kathariou S. (2018). Proximity to other commercial turkey farms affects colonization onset, genotypes, and antimicrobial resistance profiles of *Campylobacter* spp. in turkeys: Suggestive evidence from a paired-farm model. Appl. Environ. Microbiol..

[15] Smith K., Reimers N., Barnes H.J., Lee B.C., Siletzky R., Kathariou S. (2004). *Campylobacter* colonization of sibling turkey flocks reared under different management conditions. J. Food Prot..

[16] Barnes H.J., Guy J.S., Vaillancourt J.P. (2000). Poult enteritis complex. Rev. Sci. Tech..

[17] Oakley B.B., Lillehoj H.S., Kogut M.H., Kim W.K., Maurer J.J., Pedroso A., Lee M.D., Collett S.R., Johnson T.J., Cox N.A. (2014). The chicken gastrointestinal microbiome. FEMS Microbiol. Lett..

[18] Teirlynck E., Gussem M.D., Dewulf J., Haesebrouck F., Ducatelle R., Van Immerseel F. (2011). Morphometric evaluation of “dysbacteriosis” in broilers. Avian Pathol..

[19] Danzeisen J.L., Calvert A.J., Noll S.L., McComb B., Sherwood J.S., Logue C.M., Johnson T.J. (2013). Succession of the turkey gastrointestinal bacterial microbiome related to weight gain. PeerJ.

[20] Day J.M., Zsak L. (2015). Investigating turkey enteric picornavirus and its association with enteric disease in poults. Avian Dis..

[21] Feighner S.D., Dashkevicz M.P. (1987). Subtherapeutic levels of antibiotics in poultry feeds and their effects on weight gain, feed efficiency, and bacterial cholyltaurine hydrolase activity. Appl. Environ. Microbiol..

[22] Allen H.K., Stanton T.B. (2014). Altered egos: Antibiotic effects on food animal microbiomes. Annu. Rev. Microbiol..

[23] Diarra M.S., Malouin F. (2014). Antibiotics in Canadian poultry productions and anticipated alternatives. Front. Microbiol..

[24] Lammie S.L., Hughes J.M. (2016). Antimicrobial resistance, food safety, and One Health: The need for convergence. Annu. Rev. Food Sci. Technol..

[25] Cui S., Ge B., Zheng J., Meng J. (2005). Prevalence and antimicrobial resistance of *Campylobacter* spp. and *Salmonella* serovars in organic chickens from Maryland retail stores. Appl. Environ. Microbiol..

[26] El-Adawy H., Ahmed M.F., Hotzel H., Tomaso H., Tenhagen B.A., Hartung J., Neubauer H., Hafez H.M. (2015). Antimicrobial susceptibilities of *Campylobacter jejuni* and *Campylobacter coli* recovered from organic turkey farms in Germany. Poult. Sci..

[27] El-Adawy H., Hotzel H., Tomaso H., Neubauer H., Hafez H.M. (2012). Elucidation of colonization time and prevalence of thermophilic *Campylobacter* species during turkey rearing using multiplex polymerase chain reaction. Poult. Sci..

[28] Wallace J.S., Stanley K.N., Jones K. (1998). The colonization of turkeys by thermophilic campylobacters. J. Appl. Microbiol..

[29] Gharst G., Hanson D., Kathariou S. (2006). Effect of direct culture versus selective enrichment on the isolation of thermophilic *Campylobacter* from feces of mature cattle at harvest. J. Food Prot..

[30] Gu W., Siletzky R.M., Wright S., Islam M., Kathariou S. (2009). Antimicrobial susceptibility profiles and strain type diversity of *Campylobacter jejuni* isolates from turkeys in eastern North Carolina. Appl. Environ. Microbiol..

[31] Miller W.G., Englen M.D., Kathariou S., Wesley I.V., Wang G., Pittenger-Alley L., Siletz R.M., Muraoka W., Fedorka-Cray P.J., Mandrell R.E. (2006). Identification of host-associated alleles by multilocus sequence typing of *Campylobacter coli* strains from food animals. Microbiology.

[32] Miller W.G., On S.L.W., Wang G., Fontanoz S., Lastovica A.J., Mandrell R.E. (2005). Extended multilocus sequence typing system (MLST) for *Campylobacter coli*, *C. lari, C. upsaliensis* and *C. helveticus*. J. Clin. Microbiol..

[33] Hoogenboom L.A., Bokhorst J.G., Northolt M.D., van de Vijver L.P., Broex N.J., Mevius D.J., Meijs J.A., Van der Roest J. (2008). Contaminants and microorganisms in Dutch organic food products: A comparison with conventional products. Food Addit. Contam. Part A Chem. Anal. Control Expo. Risk Assess..

[34] Van Overbeke I., Duchateau L., De Zutter L., Albers G., Ducatelle R. (2006). A comparison survey of organic and conventional broiler chickens for infectious agents affecting health and food safety. Avian Dis..

[35] McCrackin M.A., Helke K.L., Galloway A.M., Poole A.Z., Salgado C.D., Marriott B.P. (2016). Effect of antimicrobial use in agricultural animals on drug-resistant foodborne Campylobacteriosis in humans: A systematic literature review. Crit. Rev. Food Sci. Nutr..

[36] Good L., Miller W.G., Niedermeyer J., Osborne J., Siletzky R.M., Carver D., Kathariou S. (2019). Strain-specific differences in survival of *Campylobacter* spp. in naturally contaminated turkey feces and water. Appl. Environ. Microbiol..

[37] Luangtongkum T., Morishita T.Y., Ison A.J., Huang S., McDermott P.F., Zhang Q. (2006). Effect of conventional and organic production practices on the prevalence and antimicrobial resistance of *Campylobacter* spp. in poultry. Appl. Environ. Microbiol..

[38] Noormohamed A., Fakhr M.K. (2014). Prevalence and antimicrobial susceptibility of *Campylobacter* spp. in Oklahoma conventional and organic retail poultry. Open Microbiol. J..

