# Supplementary material for: Campylobacter Colonization and Diversity in Young Turkeys in the Context of Gastrointestinal Distress and Antimicrobial Treatment
Source: Microorganisms. 2023 Jan 19;11(2):252. doi: 10.3390/microorganisms11020252 (PMC9963665; doi:10.3390/microorganisms11020252)
Supplement: Supplementary file 1 [file microorganisms-11-00252-s001.zip › microorganisms-2134996-supplementary.pdf]

SUPPLEMENTARY TABLES

Supplementary Table 1: *Campylobacter* content in the cecum and jejunum of individual birds

Flock 1

Timepoint	Bird	Cecum		Jejunum	
		Detection[1]	CFU/g	Detection	CFU/g
Day of hatch	All birds below detection limit				
Week 1	All birds below detection limit				
Week 2	All birds below detection limit				
Week 3	Bird 1	Positive	3.0×10^4	Positive	1.2×10^5
	Bird 2	Positive	3.5×10^4	Positive	1.7×10^5
	Bird 3	Positive	2.5×10^7	Positive	2.4×10^5
	Bird 4	Positive	3.2×10^7	Positive	2.2×10^4
	Bird 5	Positive	1.5×10^7	Positive	5.0×10^3
	Bird 6	Positive	2.1×10^8	Positive	1.8×10^3
	Bird 7	Positive	9.5×10^6	Positive	7.0×10^5
	Bird 8	Positive	1.5×10^6	Positive	1.6×10^6
	Bird 9	Positive	2.4×10^8	Positive	1.9×10^6
	Bird 10	Positive	9.4×10^6	Positive	2.2×10^4
Week 4	Bird 1	Positive	1.1×10^9	Positive	1.4×10^6
	Bird 2	Positive	1.5×10^9	Positive	1.7×10^6
	Bird 3	Positive	8.7×10^8	Positive	9.8×10^5
	Bird 4	Positive	1.6×10^9	Positive	1.0×10^5
	Bird 5	Positive	6.7×10^8	Positive	3.2×10^5
	Bird 6	Positive	5.6×10^9	Positive	3.2×10^5
	Bird 7	Positive	4.4×10^9	Positive	2.2×10^5
	Bird 8	Positive	2.6×10^9	Positive	1.4×10^6
	Bird 9	Positive	4.3×10^8	Positive	1.1×10^5
	Bird 10	Positive	9.0×10^9	Positive	9.8×10^4
Week 5	Bird 1	Positive	$4.0 x 10^8$	Positive	4.0×10^4
	Bird 2	Positive	$2.9 x 10^5$	Positive	3.5×10^6
	Bird 3	BDL	BDL	Positive	2.4×10^4
	Bird 4	Positive	$3.1 x 10^8$	Positive	3.0×10^4
	Bird 5	Positive	$2.5 x 10^8$	Positive	5.4×10^4
	Bird 6	Positive	$4.3 x 10^7$	Positive	7.5×10^4
	Bird 7	Positive	$4.5 x 10^5$	Positive	1.5×10^4
	Bird 8	Positive	$5.7 x 10^8$	Positive	2.2×10^5
	Bird 9	Positive	$4.9 x 10^7$	Positive	2.2×10^5
	Bird 10	Positive	$2.3 x 10^7$	Positive	5.0×10^3

Flock 2

Timepoint	Bird	Cecum		Jejunum	
		Detection	CFU/g	Detection	CFU/g
Day of hatch	All birds below detection limit				
Week 1	All birds below detection limit				
Week 2	Bird 1	BDL	BDL	BDL	BDL
	Bird 2	Positive	2.0×10^6	Positive	2.5×10^4
	Bird 3	Positive	BDL	BDL	BDL
	Bird 4	Positive	2.0×10^8	Positive	1.1×10^4
	Bird 5	Positive	1.3×10^5	Positive	1.0×10^4
	Bird 6	Positive	4.5×10^4	Positive	2.2×10^4
	Bird 7	BDL	BDL	BDL	BDL
	Bird 8	Positive	BDL	Positive	1.7×10^4
	Bird 9	BDL	BDL	BDL	BDL
	Bird 10	BDL	BDL	BDL	BDL
Week 3	Bird 1	Positive	1.5×10^9	Positive	1.0×10^3
	Bird 2	Positive	4.4×10^8	Positive	5.0×10^3
	Bird 3	Positive	2.3×10^7	BDL	BDL
	Bird 4	Positive	2.3×10^8	BDL	BDL
	Bird 5	Positive	6.0×10^5	Positive	1.2×10^5
	Bird 6	Positive	1.9×10^9	Positive	1.0×10^4
	Bird 7	Positive	2.5×10^9	BDL	BDL
	Bird 8	Positive	4.4×10^7	BDL	BDL
	Bird 9	Positive	4.1×10^7	Positive	7.0×10^3
	Bird 10	Positive	4.5×10^8	Positive	1.2×10^4
Week 4	Bird 1	Positive	7.0×10^4	Positive	5.2×10^4
	Bird 2	Positive	5.1×10^8	Positive	1.0×10^7
	Bird 3	Positive	1.0×10^7	Positive	2.9×10^4
	Bird 4	Positive	1.3×10^7	Positive	2.8×10^6
	Bird 5	Positive	1.5×10^9	Positive	2.5×10^4
	Bird 6	Positive	1.9×10^9	Positive	3.9×10^6
	Bird 7	BDL	BDL	Positive	2.0×10^5
	Bird 8	Positive	2.3×10^7	Positive	8.5×10^4
	Bird 9	Positive	5.6×10^7	Positive	2.0×10^6
	Bird 10	Positive	3.8×10^8	Positive	2.4×10^6

Flock 3

Timepoint	Bird	Cecum		Jejunum	
		Detection	CFU/g	Detection	CFU/g
Day of hatch	All birds below detection limit				
Week 1	All birds below detection limit				
Week 2	Bird 1	Positive	5.0×10^5	BDL	BDL
	Bird 2	BDL	BDL	BDL	BDL
	Bird 3	Positive	BDL	BDL	BDL
	Bird 4	BDL	BDL	BDL	BDL
	Bird 5	BDL	BDL	Positive	2.2×10^5
	Bird 6	Positive	4.3×10^5	BDL	BDL
	Bird 7	BDL	BDL	Positive	3.0×10^4
	Bird 8	BDL	BDL	Positive	1.9×10^5
	Bird 9	BDL	BDL	BDL	BDL
	Bird 10	BDL	BDL	BDL	BDL
Week 3	Bird 1	BDL	BDL	Positive	1.3×10^5
	Bird 2	Positive	1.0×10^7	Positive	1.2×10^6
	Bird 3	Positive	3.5×10^6	Positive	1.2×10^6
	Bird 4	Positive	1.6×10^9	Positive	7.5×10^5
	Bird 5	Positive	1.1×10^8	BDL	BDL
	Bird 6	Positive	4.4×10^9	Positive	9.0×10^4
	Bird 7	Positive	1.4×10^9	Positive	3.0×10^4
	Bird 8	Positive	2.3×10^7	Positive	8.1×10^5
	Bird 9	Positive	1.7×10^9	Positive	9.8×10^5
	Bird 10	Positive	6.5×10^4	Positive	8.7×10^5
Week 4	Bird 1	Positive	1.7×10^9	BDL	BDL
	Bird 2	Positive	4.0×10^9	Positive	5.2×10^6
	Bird 3	Positive	6.6×10^7	Positive	3.5×10^5
	Bird 4	Positive	2.5×10^5	Positive	1.1×10^6
	Bird 5	Positive	5.2×10^9	Positive	4.0×10^3
	Bird 6	Positive	1.6×10^5	Positive	4.5×10^5
	Bird 7	Positive	9.0×10^7	Positive	4.1×10^5
	Bird 8	Positive	5.0×10^4	Positive	5.6×10^4
	Bird 9	Positive	3.0×10^9	Positive	2.9×10^6
	Bird 10	Positive	5.3×10^7	Positive	4.0×10^5
Week 5	Bird 1	Positive	2.4×10^8	Positive	1.8×10^5
	Bird 2	Positive	2.7×10^8	Positive	4.5×10^4
	Bird 3	Positive	3.2×10^8	Positive	8.0×10^3
	Bird 4	Positive	1.1×10^7	Positive	5.5×10^6
	Bird 5	Positive	1.1×10^8	Positive	BDL
	Bird 6	Positive	2.4×10^8	Positive	6.3×10^5
	Bird 7	Positive	3.2×10^8	Positive	5.7×10^6
	Bird 8	Positive	1.1×10^7	Positive	4.8×10^4
	Bird 9	Positive	1.4×10^7	Positive	BDL
	Bird 10	Positive	4.2×10^8	Positive	1.9×10^5

Flock 4

Timepoint	Bird	Cecum		Jejunum	
		**Detection**	**CFU/g**	**Detection**	**CFU/g**
Day of hatch	All birds below detection limit				
Week 1	All birds below detection limit				
Week 2	All birds below detection limit				
Week 3	Bird 1	Positive	1.1×10^7	BDL	BDL
	Bird 2	Positive	8.4×10^8	Positive	2.3×10^5
	Bird 3	Positive	5.6×10^7	Positive	5.0×10^4
	Bird 4	Positive	9.9×10^8	Positive	8.3×10^5
	Bird 5	Positive	2.0×10^6	Positive	8.5×10^3
	Bird 6	Positive	1.0×10^9	Positive	2.0×10^4
	Bird 7	Positive	7.8×10^8	Positive	8.0×10^3
	Bird 8	Positive	1.2×10^5	Positive	9.7×10^5
	Bird 9	Positive	1.3×10^5	Positive	7.5×10^3
	Bird 10	Positive	4.9×10^5	BDL	BDL
Week 4	Bird 1	Positive	1.9×10^9	Positive	2.7×10^4
	Bird 2	Positive	3.0×10^9	Positive	4.8×10^5
	Bird 3	Positive	1.2×10^7	Positive	3.0×10^5
	Bird 4	Positive	5.0×10^7	Positive	3.8×10^4
	Bird 5	Positive	6.5×10^7	Positive	1.1×10^5
	Bird 6	Positive	1.8×10^7	BDL	BDL
	Bird 7	Positive	4.3×10^9	Positive	1.7×10^4
	Bird 8	Positive	2.0×10^9	Positive	1.2×10^5
	Bird 9	Positive	7.5×10^6	Positive	1.8×10^5
	Bird 10	Positive	3.1×10^7	Positive	4.3×10^4
Week 5	Bird 1	Positive	2.4×10^5	Positive	4.0×10^6
	Bird 2	Positive	2.3×10^5	Positive	5.4×10^5
	Bird 3	Positive	2.0×10^7	Positive	6.3×10^6
	Bird 4	Positive	1.8×10^8	Positive	3.2×10^5
	Bird 5	Positive	1.8×10^7	Positive	BDL
	Bird 6	Positive	2.0×10^9	Positive	2.4×10^5
	Bird 7	Positive	4.3×10^7	Positive	BDL
	Bird 8	Positive	3.4×10^8	Positive	2.1×10^4
	Bird 9	Positive	7.0×10^6	Positive	3.8×10^5
	Bird 10	Positive	2.1×10^8	Positive	3.5×10^5

[1] BDL: below detection limit.

Supplementary Table 2: *Campylobacter* isolates typed by MLST.

Isolate[1]	Species	AMR profile[2]	Date of sample	ST[3]	Origin	*aspA*	*glnA*	*gltA*	*glyA*	*pgm*	*tkt*	*uncA*
F1C1W3A	*C. coli*	TSEKQG	5/11/16	889	Cecum	33	39	30	82	113	47	41
F1C5W5D	*C. coli*	TSEKQG	5/25/16	8086	Cecum	33	39	30	82	188	43	79
F1J4W4A	*C. coli*	TEKG	5/18/16	**8532**	Jejunum	55	110	103	2	188	261	79
F1C5W4C	*C. coli*	TKG	5/18/16	**8532**	Cecum	55	110	103	2	188	261	79
F1C8W3D	*C. jejuni*	TSKQG	5/11/16	**8528**	Cecum	2	222	29	250	188	25	35
F1J1W5D	*C. jejuni*	TSKQG	5/25/16	8227	Jejunum	2	608	29	250	303	25	35
F1C10W4C	*C. jejuni*	TKQG	5/18/16	**8522**	Cecum	7	112	5	2	6	67	6
F1J7W3C	*C. jejuni*	TSKQ	5/11/16	**8528**	Jejunum	2	222	29	250	188	25	35
F1J8W5A	*C. jejuni*	TSKQ	5/25/16	1839	Jejunum	2	222	29	250	303	25	35
F1C3W3D	*C. jejuni*	TKG	5/11/16	**8524**	Cecum	7	112	287	2	6	67	6
F1C2W4A	*C. jejuni*	TKG	5/18/16	**8522**	Cecum	7	112	5	2	6	67	6
F1C1W5A	*C. jejuni*	TKG	5/25/16	**8522**	Cecum	7	112	5	2	6	67	6
F1J3W3E	*C. jejuni*	TK	5/11/16	**8522**	Jejunum	7	112	5	2	6	67	6
F2C1W3D	*C. coli*	TSEKQG	5/11/16	889	Cecum	33	39	30	82	113	47	41
F2C5W4D	*C. coli*	TSEKQG	5/18/16	1119	Cecum	33	39	30	82	113	43	41
F2C3W3C	*C. coli*	TEKQG	5/11/16	**8523**	Cecum	7	39	30	140	104	43	41
F2C2W2B	*C. coli*	TKQG	5/04/16	**8531**	Cecum	55	110	103	171	188	169	79
F2C5W3C	*C. coli*	TKQG	5/11/16	**8534**	Cecum	2	110	103	171	188	169	79
F2J10W3C	*C. jejuni*	TSKQG	5/11/16	8227	Jejunum	2	608	29	250	303	25	35
F2C4W4A	*C. jejuni*	TSKQG	5/18/16	8227	Cecum	2	608	29	250	303	25	35
F2J1W3A	*C. jejuni*	TKQG	5/11/16	**8530**	Jejunum	2	608	29	250	303	67	35
F2C2W4C	*C. jejuni*	TKQG	5/18/16	**8529**	Cecum	7	222	29	250	303	25	35
F2C3W4C	*C. jejuni*	TKQ	5/18/16	8227	Cecum	2	608	29	250	303	25	35
F3C1W2D	*C. coli*	TSEKQG	5/11/16	1604	Cecum	33	39	30	82	188	43	41
F3C1W4A	*C. coli*	TSEKQG	5/25/16	1604	Cecum	33	39	30	82	188	43	41
F3C4W5A	*C. coli*	TSEKQG	6/01/16	1604	Cecum	33	39	30	82	188	43	41
F3C5W4A	*C. coli*	TEKQG	5/25/16	**8531**	Cecum	55	110	103	171	188	169	79
F3C6W4A	*C. coli*	TEKQG	5/25/16	**8531**	Cecum	55	110	103	171	188	169	79
F3C6W4B	*C. coli*	TEKQG	5/25/16	**8531**	Cecum	55	110	103	171	188	169	79
F3C9W3C	*C. coli*	TSEQG	5/18/16	1604	Cecum	33	39	30	82	188	43	41
F3C6W2E	*C. jejuni*	TSKQG	5/11/16	1839	Cecum	2	222	29	250	303	25	35
F3J7W4A	*C. jejuni*	TSKQG	5/25/16	1839	Jejunum	2	222	29	250	303	25	35
F3C2W5A	*C. jejuni*	TSKQG	6/01/16	1839	Cecum	2	222	29	250	303	25	35
F3J2W3C	*C. jejuni*	TKQG	5/18/16	1839	Jejunum	2	222	29	250	303	25	35
F3J2W4B	*C. jejuni*	TKQG	5/25/16	8227	Jejunum	2	608	29	250	303	25	35

Supplementary Table 2 (cont.)

Isolate[1]	Species	AMR profile[2]	Date of sample	ST[3]	Origin	*aspA*	*glnA*	*gltA*	*glyA*	*pgm*	*tkt*	*uncA*
F3C7W5A	*C. jejuni*	TKQG	6/01/16	8227	Cecum	2	608	29	250	303	25	35
F3J7W3A	*C. jejuni*	TKG	5/18/16	**8527**	Jejunum	7	112	5	2	303	61	6
F4C9W3A	*C. coli*	TSEKQG	5/18/16	1604	Cecum	33	39	30	82	188	43	41
F4C6W4B	*C. coli*	TSEKQG	5/25/16	1604	Cecum	33	39	30	82	188	43	41
F4C8W5B	*C. coli*	TSEKQG	6/01/16	**8521**	Cecum	33	110	30	82	188	43	35
F4J3W4B	*C. coli*	TEKQG	5/25/16	**8531**	Jejunum	55	110	103	171	188	169	79
F4J3W4A	*C. coli*	TEKQG	5/25/16	**8531**	Jejunum	55	110	103	171	188	169	79

F4C9W5B	*C. coli*	TEKQG	6/01/16	**8531**	Cecum	55	110	103	171	188	169	79
F4C7W4B	*C. coli*	TKG	5/25/16	1192	Cecum	103	110	103	172	188	169	79
F4C1W5A	*C. coli*	TKG	6/01/16	**8533**	Cecum	55	110	103	172	188	261	79
F4J8W3C	*C. jejuni*	TSKQG	5/18/16	8227	Jejunum	2	608	29	250	303	25	35
F4J5W4A	*C. jejuni*	TSKQG	5/25/16	1839	Jejunum	2	222	29	250	303	25	35
F4C7W5B	*C. jejuni*	TSKQG	6/01/16	1839	Cecum	2	222	29	250	303	25	35
F4C5W5A	*C. jejuni*	TSKQ	6/01/16	**8542**	Cecum	7	112	5	2	303	67	6
F4J1W4A	*C. jejuni*	TKQG	5/25/16	**8542**	Jejunum	7	112	5	2	303	67	6
F4J2W4D	*C. jejuni*	TKQG	5/25/16	**8525**	Jejunum	7	110	5	2	303	67	6
F4C2W4D	*C. jejuni*	TKG	5/25/16	**8542**	Cecum	7	112	5	2	303	67	6
F4J7W5A	*C. jejuni*	TKG	6/01/16	**8526**	Jejunum	2	112	5	2	303	67	6

[1] Isolate designations consist of flock number (F1, F2 etc.), intestinal sample (C or J for cecum and jejunum, respectively), bird number (1-10), week (W1-W4 or W1-W5, depending on flock), and a letter (A-D) pertaining to the specific isolate from that sample. For instance, F1C1W3A was isolate A from the cecum of bird # 1 from flock 1, week 3 and F1J4W4A was isolate A from the jejunum of bird # 4 from flock 1, week 4.

[2] T: tetracycline; S: streptomycin; E: erythromycin; G: gentamicin; K: kanamycin; Q: (fluoro)quinolones (nalidixic acid and ciprofloxacin).

[3] Novel sequence types identified in this study are in bold.
